# Involvement of Maternal and Socioeconomic Risk Factors in the Incidence of Fetal Growth Restriction in a Large Maternity Hospital in Romania

**DOI:** 10.3390/children12020152

**Published:** 2025-01-28

**Authors:** Mariana-Lăcrămioara Bucur-Grosu, Andreea Avasiloaiei, Iolanda Valentina Popa, Luminița Păduraru, Daniela Cristina Dimitriu, Demetra Socolov

**Affiliations:** 1 “Cuza-Vodă” Clinical Hospital of Obstetrics and Gynecology, 700038 Iași, Romania; mbucurgrosu@yahoo.com (M.-L.B.-G.); luminita.paduraru@umfiasi.ro (L.P.); daniela.dimitriu@umfiasi.ro (D.C.D.); demetra.socolov@umfiasi.ro (D.S.); 2 Doctoral School, “Grigore T. Popa” University of Medicine and Pharmacy, 700115 Iași, Romania; 3 Department of Mother and Child Health, “Grigore T. Popa” University of Medicine and Pharmacy, 700115 Iași, Romania; 4 Faculty of Medicine, “Grigore T. Popa” University of Medicine and Pharmacy, 700115 Iași, Romania; iolanda-valentina.g.popa@umfiasi.ro

**Keywords:** fetal growth restriction, risk factors, pregnancy-induced hypertension, low birth weight, small for gestational age

## Abstract

**Background:** Fetal growth restriction (FGR) refers to a condition in which a fetus does not reach its genetically predetermined growth potential due to various pathological factors of maternal or fetal origin, with potential long-life consequences, such as elevated blood pressure, type 2 diabetes mellitus, obesity, dyslipidemia, atherosclerosis. **Aim:** The purpose of our research is to delve into the intricate relationship between economic and social factors and the occurrence of FGR. **Methods:** We analyzed risk factors previously associated with FGR and aimed to compare them between two cohorts of infants with FGR: a historical cohort of infants born from 2010 to 2012 and a contemporary cohort of infants born from 2020 to 2022. **Results:** The global incidence of FGR in our study was 5.13%, with non-significant differences between the two time periods: 5.03% in the historical cohort and 5.25% in the contemporary cohort. More mothers of FGR infants receive formal education and are employed in the contemporary group and thus have a more stable income. There was a major decrease in the number of preterm infants with FGR, from 23.9% in the historical cohort to 5.9% in the contemporary cohort (*p* < 0.001). Compared to the historical cohort, we found significant increases in the incidence of pregnancy-induced hypertension, Cesarean sections, and prenatal follow-up in the contemporary cohort (8.3% vs. 3.8%, *p* < 0.001; 59.2% vs. 49.9%, *p* < 0.001; 67.7% vs. 49.6%, *p* < 0.001, respectively) and we also found significant correlations between prenatal care on one side and maternal smoking, urban residence, higher maternal education, and prematurity on the other. **Conclusions:** Certain socioeconomic factors show definite improvements over the ten-year timespan, which results in an increase in prenatal care and a decrease in the rate of prematurity. However, the incidence of FGR remains constant over the considered period, meaning that other factors, apart from socioeconomic factors, play a substantial role. Recognizing these risk factors is crucial for developing effective public health strategies aimed at reducing the incidence of FGR and improving maternal and child outcomes.

## 1. Introduction

Fetal growth restriction (FGR) remains a dominant cause of morbidity both in the neonatal period and, according to the developmental origin of health and disease (DOHaD) theory, throughout one’s entire life [1,2,3]. Through the process of “fetal programming” [4], FGR is incriminated as a starting point for various non-communicable conditions, such as elevated blood pressure, type 2 diabetes mellitus, obesity, dyslipidemia, and atherosclerosis, both in later childhood and during adult life [5,6]. This represents a significant challenge to both maternal and child health, with potential long-term consequences for affected individuals.

Socioeconomic factors have been indicated in several studies to be involved in the pathogeny of low birth weight, including FGR [7]. This paper explores the current state of knowledge regarding the impact of socioeconomic factors on FGR, including maternal education, income disparities, access to healthcare, and environmental conditions, as well as selected gestational factors, while also analyzing the data in our hospital in an effort to shed light on this intricate and complex neonatal condition.

FGR refers to a condition in which a fetus does not reach its genetically predetermined growth potential due to various pathological factors, such as placental abnormalities or maternal conditions, such as arterial hypertension, obesity, or anemia [8]. These create an unfavorable intrauterine environment in which the offspring is compelled to adapt its energetic expenditures in order to survive. Small-for-gestational-age (SGA) infants, on the other hand, are those whose birth weight (BW) is below the 10th percentile for their gestational age (GA) but may not necessarily have experienced any growth restrictions—some are constitutionally small, as dictated by parental anthropometry and ethnicity, but are otherwise healthy.

In the absence of a standardized, universally accepted definition of this condition, FGR and small-for-gestational-age infants are often superimposed. The real overlap is only partially accurate since a small number of FGR have appropriate-for-gestational-age birth weights, and some SGA infants are intrinsically small, without being restricted and without the high probability of a poor outcome. However, the equivalence FGR to SGA will be kept throughout our work, the main reason being that in our region of Romania, there is a large proportion of pregnancies that do not receive adequate obstetric monitoring and thus might lack the proper diagnosis of fetal growth restriction.

## 2. Materials and Methods

### 2.1. Subjects

We performed a comparative analysis on two retrospective cohorts of infants born in our maternity hospital, a referral unit for the region of Romania with a high incidence of fetal growth restriction and high-risk pregnancies.

For this study, we used SGA as a proxy for FGR; thus, we defined FGR as birth weight (BW) below the 10th percentile on the World Health Organization Fetal Growth Charts [9].

As inclusion criteria, we used FGR (per the definition above) and singleton live pregnancies. The exclusion criteria were birth weight above the 10th percentile, twin/multiple pregnancy, severe congenital malformations, and stillborn infants.

The first cohort is the “historical cohort”, which is made up of 912 infants with FGR born over three years (2010–2012)—Group H—and the second one is the “contemporary cohort”, which contains 937 infants with FGR also born over three years (2020–2022)—Group C.

This study was approved by the university’s Research Ethics Council (no. 250/27 December 2022). Informed consent was obtained from the parents/guardians of all subjects involved in the study.

### 2.2. Data Collection

For both cohorts, we collected items we deemed influential for the prevalence of FGR (pregnancy follow-up, gestation, parity, education, occupation, smoking, living circumstances—rural/urban, maternal hypertension) in order to detect the emergence of certain patterns over the course of ten years. All information was freely provided to the attending neonatologist by the mother upon the first interview after birth. We also collected data regarding the neonates, such as sex, gestational age (GA), birth weight (BW), and length.

### 2.3. Data Preprocessing

The initial dataset contained missing values, which were handled via imputation using the k-nearest neighbors (kNN) algorithm implemented in the VIM package in R. The kNN algorithm was applied with k set to 5, which identifies the 5 nearest neighbors for each missing value based on Euclidean distance. Imputed values were assigned from these neighbors. This method allows for robust imputation by leveraging similarities within the dataset while preserving the integrity of the original data structure. This resulted in a clean dataset that was suitable for analysis.

### 2.4. Statistical Comparison Between Time Periods

To evaluate differences in demographic and clinical characteristics between the two time periods (2010–2012 and 2020–2022), we used the Wilcoxon rank sum test for numerical variables due to their non-normal distribution and the chi-square test for categorical variables. The Wilcoxon rank sum test is a non-parametric test that compares the medians of two independent groups and is suitable for non-normally distributed data. The comparison of numerical variables between the two groups is visualized using density plots. The chi-squared test assesses the association between categorical variables by comparing observed frequencies with expected frequencies under the null hypothesis of no association. The comparative analysis of the categorical variables is plotted using a cluster bar graph.

All statistical analyses were performed using RStudio 2024.04.1. The following R packages were utilized: *VIM* for data imputation, *caret* for data partitioning, *broom* for tidying model outputs, *dplyr* and *tidyr* for data manipulation, *ggplot2* for plotting, and *readxl* for data import.

## 3. Results

There was a comparable number of deliveries between the periods we analyzed (18,135 births between 2010 and 2012 and 17,851 between 2020 and 2022). The prevalence of FGR was also comparable: 5.03% (n = 912) for the historical cohort and 5.25% (n = 937) for the contemporary cohort (Table 1).

The global incidence of FGR in our study was 5.13%, with non-significant differences between the two time periods (5.03% and 5.25%, respectively).

**Figure 1:**
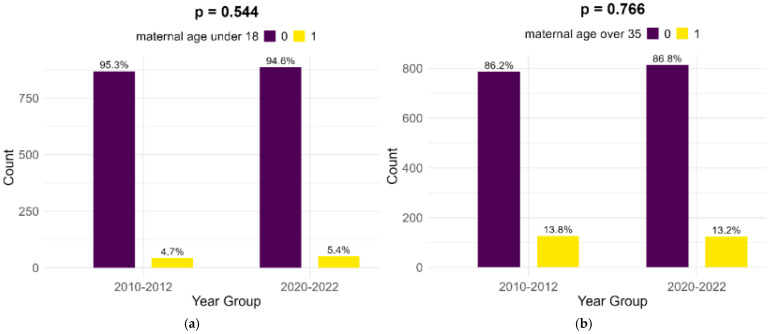
Comparison of extreme maternal ages in study cohorts: (**a**) maternal age below 18; (**b**) maternal age above 35.

Most of the mothers of infants with FGR in both cohorts were aged between 18 and 35 years (81.5% in the historical cohort and 81.4% in the contemporary cohort). Extremes of maternal age (below 18 years and above 35 years) presented no significant differences between the two cohorts, as shown in Figure 1.

**Figure 2:**
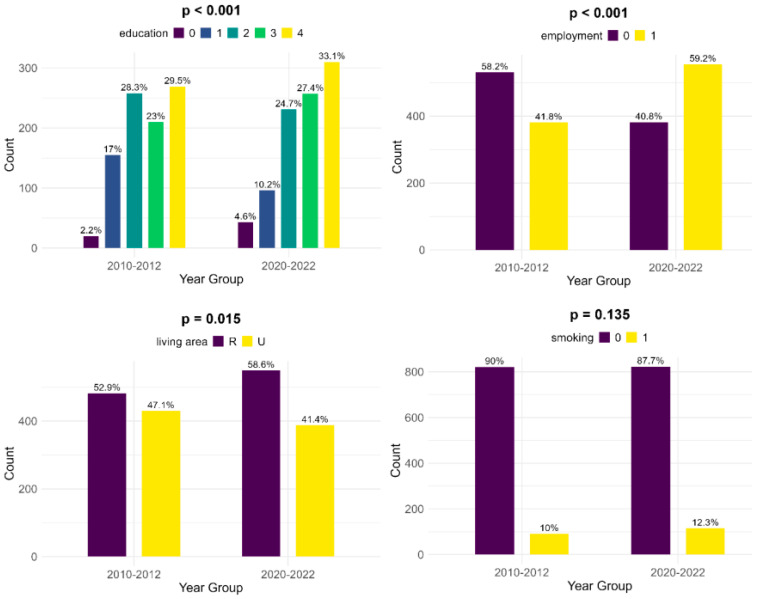
Comparison of socioeconomic risk factors between the two cohorts (upper left—education [0—no formal education, 1—primary school, 2—secondary school, 3—high school, 4—university], upper right—employment, lower left—living area [R—rural, U—urban], lower right—smoking).

The educational levels of the mothers show significant differences between the two groups: in the historical cohort, there is a lower number of women who did not receive any formal education (2.2%, compared to 4.6%). Also, mothers of infants with FGR showed less education at the primary and secondary levels in the contemporary cohort (10.2% and 24.7%, compared to 17% and 28.3%, respectively) but were more educated at the high school and university levels (27.4% and 33.1%, versus 23% and 29.5%, respectively). Compared to the historical cohort, more mothers are employed in the contemporary group and thus have a more stable income (59.2%, compared to 41.8%, *p* < 0.001). There is a lower number of women living in an urban area in the contemporary cohort (41.4% versus 47.1%, *p* = 0.015). According to our data, there was little change in smoking habits during pregnancy between the two cohorts (10% during 2010–2012 versus 12.3% during 2020–2022, *p* = 0.135) (Figure 2).

**Figure 3:**
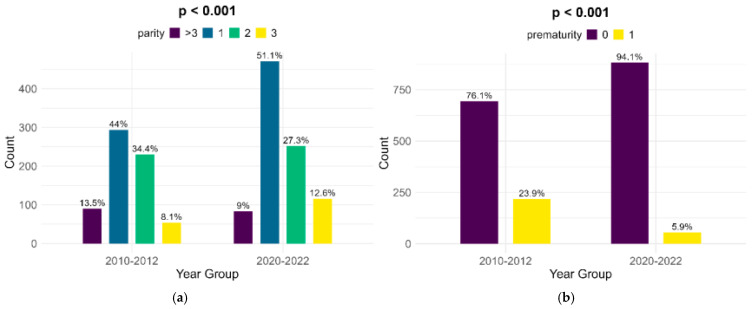
Comparison of parity (**a**) and prematurity (**b**) between the cohorts.

The parity of women with FGR infants shows significant changes between the lots, although the highest percentage of women are primiparous (44% in the historical cohort and 51.1% in the contemporary one). There was a major decrease in the number of preterm infants with FGR, from 23.9% in the historical cohort to 5.9% in the contemporary cohort (*p* < 0.001) (Figure 3).

**Figure 4:**
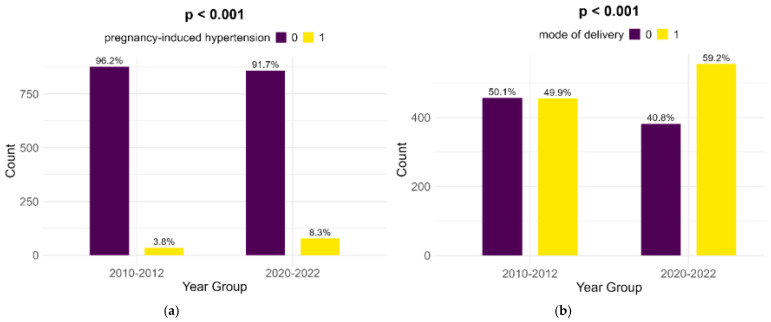
Comparison of pregnancy-induced hypertension (**a**) and mode of delivery (**b**).

Pregnancy-induced hypertension showed a significant rise from the historical cohort to the contemporary one (3.8% to 8.3%, *p* < 0.001). Also, Cesarean sections showed a highly significant increase (49.9% to 59.2%, *p* < 0.001) (Figure 4).

**Figure 5:**
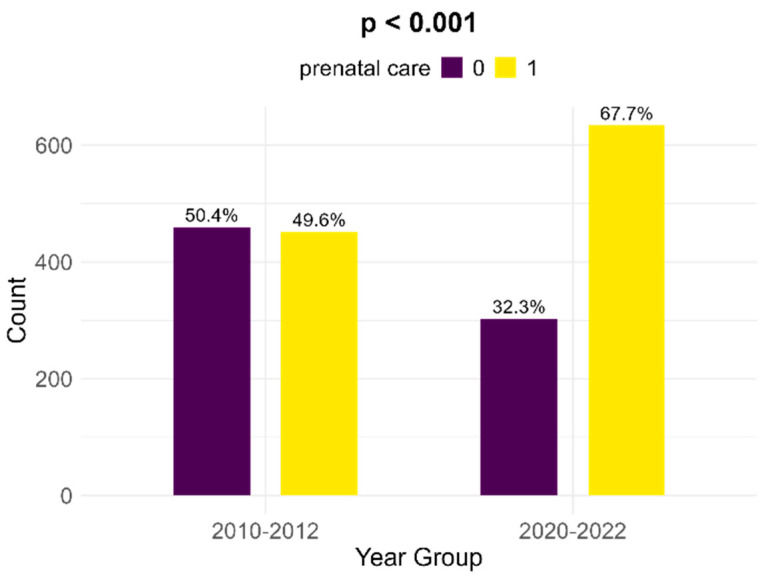
Comparison of prenatal care between the two cohorts.

Between the two cohorts, there is a significant increase in the number of women with FGR infants that received prenatal follow-up; in the historical cohort, there were 49.6% medically surveilled pregnancies, while in the contemporary cohort, 67.7% received prenatal care (*p* < 0.001, Figure 5).

For a more accurate and significant correlation between the potential determinants of FGR, we performed a bivariate analysis. Thus, we found significant correlations between prenatal care and maternal smoking, urban residence, higher maternal education, and prematurity. Also, prematurity was associated with masculine sex (53.8% versus 40.4% in term infants). There were no correlations between prematurity and maternal age below 18 years or parity versus hypertension (Table 2).

## 4. Discussion

The incidence of FGR is markedly higher in developing countries compared to more developed ones [10]. Also, the main cause of FGR in Western countries is placental insufficiency, whereas in developing societies, maternal malnutrition and infections play a more predominant role [11]. The incidence of FGR, which is between 3% and 9% in developed countries, also varies according to the population studied, fetal gestational age, and whether SGA fetuses were included [12,13].

In our study, the incidence of FGR was 5.13%. This figure is conclusive with worldwide estimates but should be considered cautiously, as it obviously does not include FGR with BW above the 10th percentile.

Several studies have highlighted the association between lower socioeconomic status and an increased risk of FGR. Notably, research by Wilding et al. emphasized the role of maternal education in influencing fetal growth while also exploring the link between income disparities and SGA prevalence in their study spanning over 13 years [14]. Additionally, the review by Veras et al. shed light on the impact of environmental factors, such as air pollution and living circumstances, on fetal development [15]. Similar results, linking air pollution, but not noise, to FGR, were obtained in two urban areas in Dijon, France [16].

In our study, the level of maternal education has changed from the historical cohort to the contemporary cohort; in the latter, mothers of infants with FGR exhibit lower percentages for any education, as well as primary and secondary education, but higher numbers when it comes to higher education (high school and university). The higher number of mothers residing in a rural area may also impact school availability and attendance.

Maternal education is linked to prenatal care—we found an extremely significant association between advanced maternal education and prenatal care in both time frames, which is in line with previously reported data [17]. Prenatal care is, in turn, associated with women from urban environments, which is probably due to easier access to medical facilities; this change over 10 years may be attributed to people moving to the suburban areas surrounding the main cities.

An interesting study from Brazil compared two groups of infants with intrauterine growth restriction, both of which were from an urban environment but situated at opposite ends of the socioeconomic spectrum. The researchers identified a similar ratio of FGR infants but different underlying causes; on the one hand, those in the “poorer” city displayed lower income, lower education level, and lower maternal age as prominent risk factors, while in the ”wealthier” city, the main issues leading to FGR were linked to maternal smoking and a higher rate of obstetric interventions [18].

Our study shows improvement during the ten years between the periods we analyzed regarding employment; more women have stable jobs and stable incomes in the contemporary cohort compared to the historic cohort. Unfortunately, there is little change regarding smoking during pregnancy in the considered time frame, even a small, non-significant increase in the number of smokers. Also, smoking was shown to have a significant inverse correlation to prenatal care.

One surprising correlation that we found is between prenatal care and prematurity—the latter was found in 17.9% of medically surveilled pregnancies, compared to 10.4% in pregnancies in which the mothers did not receive prenatal care. This can be either iatrogenic prematurity as a result of excessive obstetric interventions (similar to the article cited above) or prematurity as a result of pregnancy-related conditions that require surveillance.

Maternal age is known as a significant risk factor for FGR. In particular, extreme ages, such as below 18 and above 35, pose a significant risk for impaired fetal growth [19]. Our study showed unremarkable changes in maternal age during the selected time frame and even a small, non-significant increase in the percentage of teenage mothers, which is inherently linked to FGR.

Parity has previously been associated with the delivery of SGA infants, as well as maternal age. A meta-analysis of 14 cohort studies showed that primiparous women, as well as those aged less than 18 years old, are at the highest risk of delivering an SGA infant [20]. Similarly, a study on more than 600,000 very small-for-gestational-age (with a BW below the fifth percentile) infants in Quebec, Canada, found an odds ratio of 1.96 for primiparous women to deliver such an infant, compared to multiparous women [21].

Our results show a peculiar dynamic of parity in the two lots; there was an increase in the number of primiparous women and women giving birth for the third time and a decrease in women having a second baby or multiparous women. The primiparous women were always the most commonly observed during both time periods, which is in line with the research mentioned above.

Pregnancy-induced hypertension (PIH) is a known and widely explored risk factor for FGR, which shows a worldwide increase in prevalence. Our research shows a significant increase in PIH over the 10-year time span, which is in line with current findings [22] and probably due to advanced maternal age, among other factors.

PIH and obesity are intrinsically linked, but while PIH is a well-known maternal risk factor for FGR, the issue concerning obesity is much more nuanced; some research recognizes the link between obesity and low birth weight [23], while other studies either do not confirm this association or link maternal obesity to the child’s higher BW [24]. The potential for bias is also significant in studies concerning maternal obesity, as they often do not mention considering gestational weight gain. In most cases, the long-term outcome of the offspring leans toward adult metabolic syndrome [25]. Although maternal obesity may very well stem from socioeconomic factors, it was not appropriately documented in our study, which made it impossible for us to draw any conclusions on the matter.

Lastly, but perhaps most relevant to our study, prenatal care has seen a dramatic increase over the past ten years, which is linked to higher maternal education and the urban residence of the mother, thus indicating better access to and understanding of life-saving medical information.

### Strengths and Limitations

One weakness of our study, which possibly accounts for the differences in the prevalence of cases compared to other studies, is the overlapping of FGR and SGA due to the fear that if we were only to consider pregnancies in which the mothers received prenatal care and a proper diagnosis of FGR, we would miss on an important percentage of subjects who had the social and economic risk factors we aimed to analyze. This limitation can be viewed as a potential strength due to the objective weight limit for each gestational age, ensuring that mistakes at inclusion were kept at a minimum.

We purposely excluded two significant socioeconomic risk factors from our analysis that could have given us further insight into this condition, alcohol consumption and marital status, due to potential misunderstandings and the societal stigma regarding both these issues, which could have prevented the interviewed women from giving honest answers.

The implications of our findings extend beyond the academic discourse, urging policymakers and healthcare providers to prioritize interventions that address socioeconomic inequities to reduce the burden of FGR. By understanding the manner in which these factors act, targeted strategies can be developed to mitigate their impact on fetal development.

## 5. Conclusions

Our findings suggest a compelling association between lower socioeconomic status and an increased likelihood of FGR. Maternal education and employment remain consistent predictors, with lower educational attainment and lack of a stable income linked to higher rates of FGR. Furthermore, environmental factors and lack of prenatal care exhibited significant correlations, emphasizing the need for a comprehensive approach to address the multifactorial nature of FGR.

There are definite improvements over the ten-year time span regarding certain socioeconomic factors, such as maternal employment and education, which resulted in an increase in prenatal care and a decrease in the rate of prematurity. Despite these dynamics, the overall prevalence of FGR remains high, meaning that economic and social factors are a minority of issues to be tackled regarding this condition. Smoking is still prevalent in mothers of SGA infants, and the percentage of teenage mothers of high-risk infants is concerning, at the least. This might represent a signal to healthcare policymakers to create campaigns aimed at deterring adolescents from drug/cigarette/vape use. Pregnancy-induced hypertension and obesity are also risk factors that may be changed with appropriate health policies concerning dietary habits and periodic check-ups at the primary healthcare provider.

Our research contributes to the evolving dialogue on the determinants of fetal growth restriction by providing a comprehensive analysis of the influence of socioeconomic factors in a hospital from the region with one of the largest prevalences of FGR in our country. Recognizing these associations and their trends is crucial for developing effective public health strategies aimed at reducing the incidence of FGR and improving maternal and child outcomes.

## Figures and Tables

**Table 1:** Comparison of cohorts.

	Group H (2010–2012)	Group C (2020–2022)	*p*
Total neonates	18,135	17,851	-
SGA (n; %)	912; 5.03%	937; 5.25%	0.446
GA (weeks)—median (IQR)	39 (37–40)	39 (38–40)	0.003
BW (grams)—median (IQR)	2500 (2150–2650)	2650 (2530–2790)	<0.001
L (cm)—median (IQR)	48 (46–49)	48 (47–49)	0.634
M/F ratio	398/514	386/551	0.3094

SGA—small-for-gestational-age; GA—gestational age; IQR—interquartile range; BW—birth weight; L—length; M/F—masculine/feminine.

**Table 2:** Bivariate analysis of risk factors for FGR.

	Pearson’s Chi-Squared Test	*p*
Prenatal care vs. smoking	X squared = 60.331; df = 1	<0.001
Prenatal care vs. living conditions	X squared = 180.41; df = 1	<0.001
Prenatal care vs. education	X squared = 784.67; df = 4	<0.001
Prenatal care vs. prematurity	X squared = 20.344; df = 1	<0.001
Prematurity vs. maternal age < 18	X squared = 0.61188; df = 1	0.434
Prematurity vs. masculine sex	X squared = 16.633; df = 1	<0.001
Parity vs. hypertension	X squared = 6.5847; df = 3	0.086

Abbreviations: df = degrees of freedom.

## Data Availability

The original contributions presented in this study are included in the article. Further inquiries can be directed to the corresponding author.

## Abbreviations

The following abbreviations are used in this manuscript:

BW	Birth weight
FGR	Fetal growth restriction
PIH	Pregnancy-induced hypertension
SGA	Small-for-gestational-age
